# Hints for the Sick-Room

**Published:** 1888-09-01

**Authors:** M. M. Basil

**Affiliations:** Author of "The Commoner Accidents to Life and Limb."


### Hints for the Sick-room.

By M. M. Basil, M.A., M.B., C.M., Author of "The Commoner Accidents to Life and Limb."

ADMINISTRATION OF MEDICINES (*continued*).

Should a medicine produce very sudden or violent effects, of which you have not been warned, report the fact at once to your superiors. If there is to be much delay before assistance can reach you, promote vomiting by giving half to a table spoonful or more of mustard flour in plenty of warm water. The immediate treatment of some of the quickly-acting poisons will be considered later on.

In all cases where a medicine has been ordered, the fact that the patient has not taken it should be reported; the nurse's omission to give it to the patient should also be duly reported. Lastly, it would be wise not to undertake the responsibility of giving any powerful medicine except when acting under an order duly signed. That which is legally an accident under one set of circumstances, may assume a very ugly form, and amount to a crime in the eye of the law, under conditions apparently similar.

Powders of small bulk may be placed on the back of the tongue, and left there to find their way into the stomach· These compact powders are particularly useful in cases where the patient is deeply insensible. Powders of large bulk must be mixed thoroughly in a tablespoon with a little water; and more water may be added after the powder has been made into a paste. The sediment must be swallowed as well as the portion held in suspension. Powders may also be given very easily in wafers or starch paper. Every portion of it must be quickly wrapped in the moistened paper, and the bolus introduced on a teaspoon into the back of the mouth, and there tumbled out of the spoon. To effect this with despatch, the spoon must be moistened, so as to prevent the starch paper adhering to it.

Pills which are or too large or too small size cannot be swallowed with comfort. The large ones cause inconvenience in their passage down the gullet; the small ones elude the grasp of the muscles of deglutition. You should report to the doctor the fact of their being badly made, since the size, form, and consistence of a pill have no necessary connexion with the medicinal ingredients contained in it, and are often left to the discretion of the chemist. Most people can be taught to swallow pills easily. In cases of extreme nervousness, bread crumbs made into pills may be employed as useful objects for exercise in this branch of education. The scenting of pills and powders is not only unnecessary, but a mischievous refinement. The taste of the drugs used for this purpose is anything but an improvement on the original evil which is supposed to be mitigated by the process. Pills may be easily given to children in rice paper.

You should be certain that a pill placed in the patient's mouth is really swallowed by him. Some retain it in the mouth and throw it away as soon as you leave them, others have been known to make a collection of them, and then to swallow several at the same time, with the intention of poisoning themselves. You should therefore habitually get the patient to speak to you before you leave him, and be certain that there is nothing in the mouth hampering his speech.

Suppositories contain medicines which are meant to act either locally on the bowel or constitutionally through the bowel. Their introduction must be effected with some care. The index finger of the right hand is generally used for this purpose. The nail must be cut short, but not to the quick—no nurse's nail must be pared to the quick. The space under the nail is best filled with some soap, and then the finger well oiled. If the patient can be turned to one or other side it is best to do so, but this is not essential. The position on the left side is the most convenient one. The patient is then asked to bear down, and the suppository introduced and followed up with the finger, the point of which is directed backwards, upwards, and to the left. The act of bearing down facilitates the operation by placing the parts in a suitable condition; it also gives the patient something to think about and prevents the attention being directed to every manœuvre, however painless in itself. If there is any spasm or colic after a suppository is introduced, you must press the nates together or support the parts through a folded napkin until the spasm is over. It is important to remember the exact direction in which the finger should be moved, as otherwise unnecessary pain will be caused in the attempt to overcome supposed obstruction. Further, it is necessary to bear in mind that if the nozzle of a hard tube or other body is to be introduced for several inches into the bowel, the direction given to the tube must be changed so as to suit the curve of the sacrum or posterior pelvic wall, which encroaches forwards into the pelvis the higher one traces it. The lower end of the tube must therefore be pushed back in proportion as its nozzle recedes from the hand.

Some medicines are given conveniently by hypodermic injection. You are not likely to be called upon to undertake this method of administering medicines until you have had considerable experience in nursing. You should, however, learn the process during your training, as cases may arise in private nursing where the periodic repetition of the operation must be left in the hands of the nurse. The procedure is very simple, and must be learned in the wards. We shall consider here mainly the negative portions of your duty in giving a hypodermic injection—in other words, I shall tell you what must *not* be done.

Before commencing the operation, see that the hypoderimc syringe is in working condition. Test its capability of sucking up some warm carbolic acid lotion, and also of ejecting it through the needle. The point of the needle must be sharp, and its shaft free from rustiness. No air must be injected under the skin. A small bubble of air will often remain in the syringe in spite of all reasonable attempts to dislodge it. If this bubble is found next the piston, or in other words, if it is not near the needle, it can do no harm, provided the piston is not driven home. No foreign matter must be introduced into the tissues; therefore, not only the needle itself must be clean, but the skin through which its point is to pierce must be equally clean. You can easily understand that unless the seat of puncture is made aseptic, there is no certainty that we do not carry into the tissues at the point of the needle specks of infectious material from the outer surface of the skin. The injection is generally made into the loose tissues under the skin, not into the skin itself. The surface of the needle must therefore be wiped perfectly dry just before its introduction, otherwise the solution moistening it will be lodged in the skin, and produce irritation, or even a small abscess. The region of the body where the injection must be made depends upon circumstances. Generally the trunk should be avoided. The flexor aspects of the limbs are the most convenient positions; the tissues here are softer than on the outer aspects. Further, the tissues of the arm are more lax than those of the forearm. But the larger arterial trunks lie mostly on the flexor aspects, and some care will be required at your hands to avoid them. The superficial veins can be easily seen and kept out of the reach of danger. The needle must be introduced with a sharp, quick, firm action of the hand. A fumbling, timorous boring into the tissues causes more pain and prolongs the nervous suspense. No unnecessary force must be used, otherwise the point of the needle may be snapped. After piercing the skin the needle is directed obliquely under it, not vertically into the parts, and it must be felt free in the subcutaneous tissues before the piston is pressed down.

(*To be continued.*)

